# Rivaroxaban thromboprophylaxis in ambulatory patients with pancreatic cancer: Results from a pre‐specified subgroup analysis of the randomized CASSINI study

**DOI:** 10.1002/cam4.3269

**Published:** 2020-07-14

**Authors:** Saroj Vadhan‐Raj, Mairéad G. McNamara, Marino Venerito, Hanno Riess, Eileen M. O'Reilly, Michael J. Overman, Xiao Zhou, Ujjwala Vijapurkar, Simrati Kaul, Peter Wildgoose, Alok A. Khorana

**Affiliations:** ^1^ The UT MD Anderson Cancer Center Department of Sarcoma Medical Oncology Section of Cytokines and Supportive Oncology Houston TX USA; ^2^ Department of Medical Oncology The Christie NHS Foundation Trust & Division of Cancer Sciences University of Manchester Manchester United Kingdom; ^3^ Klinik für Gastroenterologie Hepatologie und Infektiologie Universitätsklinikum Magdeburg Germany; ^4^ Charité Universitätsmedizin Berlin Berlin Germany; ^5^ Memorial Sloan Kettering Cancer Center Weill Cornell Medical College New York NY USA; ^6^ The UT MD Anderson Cancer Center Houston TX USA; ^7^ Janssen Research & Development, LLC Raritan NJ USA; ^8^ Janssen Scientific Affairs, LLC Titusville NJ USA; ^9^ Department of Hematology and Medical Oncology Cleveland Clinic Cleveland OH USA

**Keywords:** major bleeding, pancreatic cancer, rivaroxaban, thromboprophylaxis, venous thromboembolism

## Abstract

**Background:**

Pancreatic cancer patients are at risk for venous thromboembolism (VTE); the value of thromboprophylaxis has not been definitively established.

**Methods:**

This trial randomized cancer patients initiating a new regimen and at high risk for VTE (Khorana score ≥2) to rivaroxaban 10 mg or placebo up to day 180. This analysis examined the subset of pancreatic cancer patients. The primary efficacy endpoint was the composite of symptomatic deep‐vein thrombosis (DVT), asymptomatic proximal DVT, any pulmonary embolism, and VTE‐related death. The primary safety endpoint was International Society on Thrombosis and Haemostasis–defined major bleeding.

**Results:**

In total, 49/1080 (4.5%) patients enrolled had baseline VTE on screening, with higher rates (24/362 [6.6%]) in pancreatic cancer and they were not randomized. Of 841 randomized patients, 273 (32.5%) had pancreatic cancer; 155/273 (57% in each arm) completed the double‐blind period. The primary endpoint occurred in 13/135 (9.6%) patients in the rivaroxaban group and in 18/138 (13.0%) in the placebo group (hazard ratio [HR] = 0.70; 95% CI, 0.34‐1.43; *P* = .328) in up‐to‐day‐180 period and 5/135 (3.7%) patients receiving rivaroxaban and 14/138 (10.1%) receiving placebo in the intervention period (HR = 0.35; 95% CI, 0.13‐0.97; *P* = .034). Major bleeding was similar (2 [1.5%] receiving rivaroxaban and 3 [2.3%] receiving placebo). Correlative biomarker studies demonstrated significant decline in D‐dimer (weeks 8 and 16) in patients randomized to rivaroxaban compared to placebo (*P* < .01).

**Conclusions:**

In ambulatory pancreatic cancer patients, rivaroxaban did not result in significantly lower incidence of VTE or VTE‐related death in the 180‐day period. During the intervention period, however, rivaroxaban substantially reduced VTE without increasing major bleeding, suggesting benefit of rivaroxaban prophylaxis in this setting.

Trial registration: ClinicalTrials.gov identifier, NCT02555878.

## Graphical abstract

Thromboprophylaxis with rivaroxaban is safe and effective in the prevention of venous thromboembolism in ambulatory patients with pancreatic cancer receiving systemic therapy, with a low number needed to treat. Given the favorable risk‐benefit ratio and convenience of the oral route of administration, these findings should inform guideline recommendations for high‐risk patients.

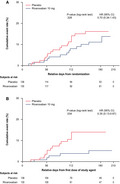

## 1 INTRODUCTION

Risk of venous thromboembolism (VTE) is substantially increased in cancer patients, resulting in increased morbidity and mortality.^1^, ^2^ The risk is further increased in cancer patients undergoing systemic therapies.^3^ Pancreatic cancer is associated with one of the highest risks of VTE of all cancers, with a cumulative risk of approximately 20%. Prognosis of patients with advanced pancreatic cancer is poor; VTE confers a worse prognosis.^4^

Although the role of anticoagulation is well established in the treatment of VTE and for the prevention of VTE in hospitalized patients, routine thromboprophylaxis of ambulatory cancer patients receiving chemotherapy is not yet recommended by guidelines due to limited evidence on efficacy and potential risk of bleeding.^5^, ^6^ Several trials have evaluated the role of thromboprophylaxis with low‐molecular‐weight heparin (LMWH) in ambulatory cancer patients receiving chemotherapy.^7^, ^8^ Although results of these trials have shown an approximate 50% reduction in the relative risk of VTE, the absolute difference was too low to recommend thromboprophylaxis in ambulatory cancer patients. Furthermore, there are limited data on safety and efficacy with novel oral anticoagulants in this setting.

Based on these findings, recently published studies have evaluated the role of direct oral anticoagulants (DOACs) for the prevention of VTE, focusing on cancer patients at intermediate to high risk for VTE (Khorana score ≥2).^9^ The results of these trials demonstrated that thromboprophylaxis with DOACs was safe and effective. Pancreatic cancer patients are at high risk for VTE, so to evaluate benefit vs risk of thromboprophylaxis in this high‐risk setting, patients were stratified into those with pancreatic cancer vs other cancers in the CASSINI trial.^10^ The results of a subgroup analysis of rivaroxaban thromboprophylaxis vs placebo in patients with pancreatic cancer are reported here.

## 2 METHODS

### 2.1 Study design and participants

The CASSINI study was a randomized, double‐blind, placebo‐controlled, multicenter trial. The design and methods of this study have been reported recently in detail.^9^, ^10^, ^11^ The study was designed by the Steering Committee members. The trial was performed in accordance with the principles of the Declaration of Helsinki and with local regulations. The protocol was approved by the institutional review board at each site. All patients provided written informed consent prior to participation.

Patients with malignancies, aged ≥18 years, initiating a new cancer regimen, and at high risk for VTE (Khorana score ≥2) were enrolled and screened with a bilateral lower‐extremity (BLE) venous duplex compression ultrasonography (CUS) to exclude pre‐existing proximal deep‐vein thrombosis (DVT).

### 2.2 Randomization and masking

Patients without thrombosis were randomly assigned 1:1 to rivaroxaban (XARELTO^®^; Janssen) 10 mg or placebo orally once daily for up to day 180 (±3 days). The randomization was stratified into tumor types of pancreas and non‐pancreas. The patient visits occurred at weeks 8 and 16 (±7 days) and on day 180 (±3 days) and included screening for DVT by BLE CUS, and routine blood tests and samples were stored for biomarker (D‐dimer, soluble P‐selectin, and tissue factor [TF]/Factor III) analysis.

### 2.3 Outcomes

The primary efficacy endpoint was a composite of objectively confirmed symptomatic or asymptomatic lower‐extremity proximal DVT, symptomatic upper‐ or lower‐extremity distal DVT, symptomatic or incidental pulmonary embolism (PE) and VTE‐related death. Secondary efficacy endpoints included components of the primary endpoint, including symptomatic VTE and clinically relevant events not included in the primary composite, such as confirmed arterial thromboembolism, confirmed visceral thromboembolism, and all‐cause mortality.

The primary safety endpoint was International Society on Thrombosis and Haemostasis–defined major bleeding (requiring transfusion or drop in hemoglobin >2 g/dL). All endpoints were adjudicated by blinded independent committees. Analyses for efficacy endpoints were conducted for the intent‐to‐treat (ITT) population for the up‐to‐day‐180 observation period and the intervention period. Safety analyses were conducted for the intervention period (time from first dose date through last dose date +2 days), only for patients who received at least one dose of study drug.

### 2.4 Biomarker data

All biomarker assays were performed by Covance Central Laboratory Services. Assays for TF and soluble P‐selectin were performed on frozen EDTA plasma, and D‐dimer levels were measured on frozen citrated plasma.

### 2.5 Statistical analysis

All efficacy analyses were based on the ITT analysis population, comprising all randomized subjects with data from randomization through day 180. In addition, analysis of the primary efficacy composite endpoint was also performed for the ITT analysis population, but for the intervention period.

Analyses of the safety endpoints related to bleeding were conducted using the same methods described for the efficacy endpoint. Bleeding endpoints were based on the safety population. The biomarker data were summarized using descriptive statistics, and treatment comparisons were performed using the Wilcoxon rank sum test. Because the occurrence of VTE and treatment of VTE can affect their levels, the biomarker data were summarized by treatment group in patients who experienced a VTE event and in those who did not. Univariate and multivariable Cox models were fitted to determine the predictive factors for a VTE event and for mortality. All statistical analyses were performed with SAS software, version 9.4.

## 3 RESULTS

### 3.1 Patients

Of 1080 patients enrolled in the CASSINI study at 143 centers in 11 countries, 362 patients had pancreatic cancer.^10^ Twenty‐four of the 362 (6.6%) patients were found to have baseline lower‐extremity DVT on screening and were not randomized. Overall, of 841 patients randomized, 273 (32.5%) had pancreatic cancer and were analyzed for efficacy; 261 patients received at least one dose of the study drug and were included in the safety analyses (Figure S1). The baseline characteristics of the patients were generally well balanced except that more patients randomly assigned to the rivaroxaban arm had a prior history of VTE, had an Eastern Cooperative Oncology Group performance status (ECOG PS) of ≥2, and were receiving at least third‐line treatment (Table 1). All except for 2 (99.3%) patients were receiving cytotoxic chemotherapy.

**Table 1:** Baseline characteristics of the study population

	Placebo	Rivaroxaban	Total
Randomized, n	138	135	273
Age, years			
Median (range)	65.0 (39.0‐87.0)	66.0 (41.0‐87.0)	66.0 (39.0‐87.0)
Gender, n (%)			
Male	77 (55.8%)	79 (58.5%)	156 (57.1%)
Female	61 (44.2%)	56 (41.5%)	117 (42.9%)
Race or ethnic group, n (%)			
White	122 (88.4%)	111 (82.2%)	233 (85.3%)
Black	4 (2.9%)	5 (3.7%)	9 (3.3%)
Asian	2 (1.4%)	3 (2.2%)	5 (1.8%)
Other	1 (0.7%)	2 (1.5%)	3 (1.1%)
Not reported	9 (6.5%)	14 (10.4%)	23 (8.4%)
Khorana risk score, n (%)			
2	100 (72.5%)	96 (71.1%)	196 (71.8%)
≥3	38 (27.5%)	39 (28.9%)	77 (28.2%)
Stage of cancer, n (%)^a^			
Stages I or II	20 (14.5%)	28 (20.7%)	48 (17.6%)
Stage III	24 (17.4%)	19 (14.1%)	43 (15.8%)
Stage IV	89 (64.5%)	82 (60.7%)	171 (62.6%)
ECOG PS score, n (%)			
0‐1	131 (94.9%)	126 (93.3%)	257 (94.1%)
≥2	6 (4.4%)	9 (6.7%)	15 (5.5%)
Prior venous thromboembolism, n (%)			
DVT	1 (0.7%)	3 (2.2%)	4 (1.5%)
PE	0 (0.0%)	1 (0.7%)	1 (0.4%)
Line of systemic cancer therapy, n (%)^b^			
First line of treatment	101 (73.2%)	97 (71.9%)	198 (72.5%)
Second line of treatment	22 (15.9%)	20 (14.8%)	42 (15.4%)
Third line of treatment	4 (2.9%)	8 (5.9%)	12 (4.4%)
Other	3 (2.2%)	4 (3.0%)	7 (2.6%)
Category of agent, n (%)			
5‐FU–based	67 (48.6%)	63 (46.7%)	130 (47.6%)
Gemcitabine‐based	61 (44.2%)	61 (45.2%)	122 (44.7%)
Gemcitabine + capecitabine/5‐FU	6 (4.3%)	7 (5.2%)	13 (4.8%)
Other cytotoxic	2 (1.4%)	3 (2.2%)	5 (1.8%)
Other noncytotoxic	1 (0.7%)	1 (0.7%)	2 (0.7%)
Unknown	1 (0.7%)	0 (0.0%)	1 (0.4%)
Laboratory values, median (range)			
D‐dimer, μg/mL^c^	1.05 (0.20‐11.71)	1.07 (0.20‐33.00)	1.07 (0.20‐33.00)
P‐selectin, ng/mL^d^	39.83 (32.00‐113.22)	40.29 (32.00‐122.82)	40.12 (32.00‐122.82)
Tissue factor, pg/mL^e^	64.00 (31.70‐280.90)	67.25 (31.90‐336.20)	65.55 (31.70‐336.20)

Abbreviations: 5‐FU, 5‐fluorouracil; DVT, deep‐vein thrombosis; ECOG PS, Eastern Cooperative Oncology Group performance status; PE, pulmonary embolism.

^a^ Tumor Node Metastasis/Ann Arbor staging.

^b^ Includes therapies that were reported in the electronic case report form at the time of randomization as well as those that were started ≤7 days from start of study agent. Note that 14 patients did not have the line of therapy denoted or had data that fell outside of this timing.

^c^ Placebo, n = 127; rivaroxaban, n = 127; overall, N = 254.

^d^ Placebo, n = 134; rivaroxaban, n = 130; overall, N = 264.

^e^ Placebo, n = 130; rivaroxaban, n = 128; overall, N = 258.

The median duration of treatment was 151 days (range, 3.0‐199), shorter in the placebo arm (128 days) than in the rivaroxaban arm (163.5 days); 57% of patients in both arms completed the double‐blind period. A higher proportion of patients in the placebo arm permanently discontinued study treatment compared with the rivaroxaban arm (58.0% vs 50.0%); reasons for discontinuation were generally similar, except a higher proportion of patients in the placebo arm discontinued due to adverse events (8.4% vs 3.1%) and study endpoint (23.7% vs 13.8%).

### 3.2 Efficacy outcomes

**Figure 1:**
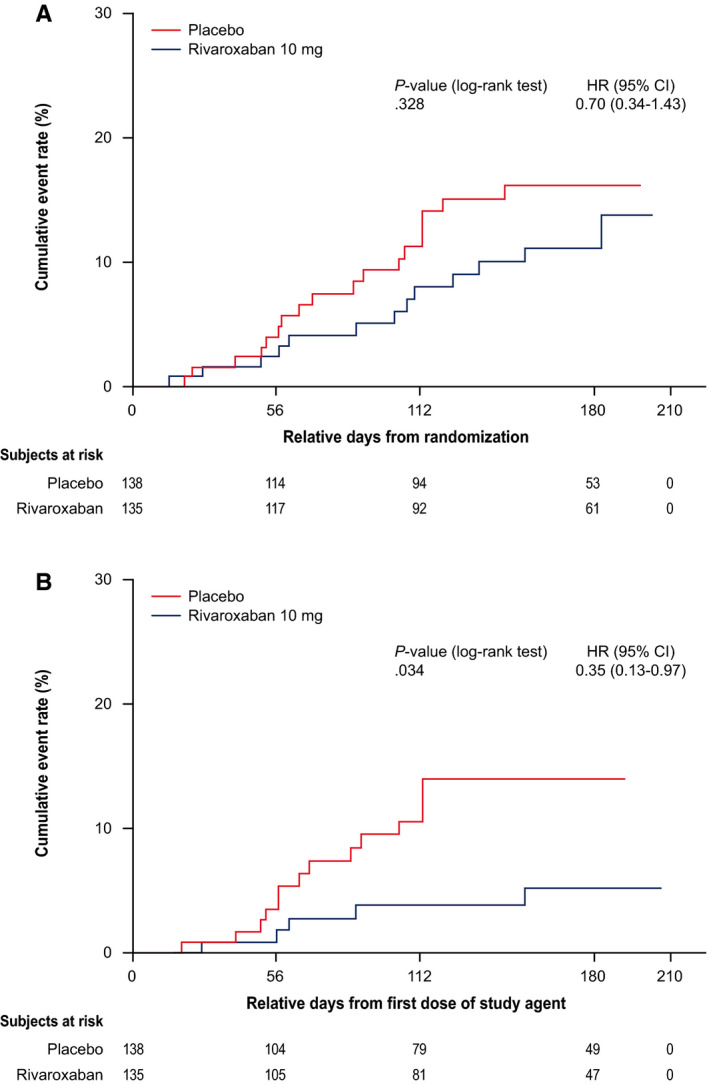
Effects of rivaroxaban vs placebo treatment on the primary composite outcome (A) at day 180 and (B) during the Intervention period.^a^ HR, hazard ratio; CI, confidence interval. ^a^The primary efficacy analyses were based on this population with data from randomization through day 180. The intervention period (on‐treatment analysis) includes all data from the first dose of study drug agent to 2 days after the last dose of the study agent

The primary efficacy composite endpoint occurred in 13/135 (9.6%) pancreatic cancer patients in the rivaroxaban group compared with 18/138 (13.0%) in the placebo group (hazard ratio [HR] = 0.70; 95% CI, 0.34‐1.43; *P* = .328; number needed to treat [NNT] = 29) in the up‐to‐day‐180 observation period (Figure 1A and Table 2). The majority of these events occurred after discontinuation of rivaroxaban compared to placebo (62% [8/13] vs 22% [4/18]). During the intervention period (Figure 1B and Table 2), the primary efficacy endpoint occurred in 5 of 135 (3.7%) patients in the rivaroxaban group compared with 14 of 138 (10.1%) patients in the placebo group (HR = 0.35; 95% CI, 0.13‐0.97; *P* = .034; NNT = 16). A breakdown of the primary endpoint for both observation periods is shown in Table 2. No heterogeneity of treatment effect was observed for any pre‐defined subgroups (*P* > .1 for all).

**Table 2:** Primary efficacy outcomes for patients with pancreatic cancer during up‐to‐day‐180 observation period and during the intervention period^a^

	Up‐to‐day‐180 observation period			Intervention period		
Outcome	Placebo (N = 138)	Rivaroxaban (N = 135)	Hazard ratio (95% CI)	Placebo (N = 138)	Rivaroxaban (N = 135)	Hazard ratio (95% CI)
Primary efficacy composite endpoint	18 (13.0%)	13 (9.6%)	0.70 (0.34‐1.43)^b^	14 (10.1%)	5 (3.7%)	0.35 (0.13‐0.97)^c^
Symptomatic^d^	9 (6.5%)	5 (3.7%)		6 (4.3%)	1 (0.7%)	
Symptomatic lower‐extremity proximal DVT	4 (2.9%)	2 (1.5%)	0.52 (0.09‐2.83)	2 (1.4%)	0 (0.0%)	NA
Symptomatic lower‐extremity distal DVT	2 (1.4%)	0 (0.0%)	NA	1 (0.7%)	0 (0.0%)	NA
Symptomatic upper‐extremity DVT	3 (2.2%)	2 (1.5%)	0.69 (0.11‐4.10)	3 (2.2%)	1 (0.7%)	0.33 (0.03‐3.18)
Symptomatic nonfatal PE	3 (2.2%)	2 (1.5%)	0.71 (0.12‐4.24)	0 (0.0%)	0 (0.0%)	NA
Asymptomatic^d^	10 (7.2%)	8 (5.9%)		9 (6.5%)	4 (3.0%)	
Asymptomatic lower‐extremity proximal DVT	5 (3.6%)	3 (2.2%)	0.57 (0.14‐2.41)	5 (3.6%)	2 (1.5%)	0.40 (0.08‐2.04)
Incidental PE	7 (5.1%)	6 (4.4%)	0.84 (0.28‐2.50)	4 (2.9%)	2 (1.5%)	0.48 (0.09‐2.60)
VTE‐related death	1 (0.7%)	0 (0.0%)	NA	0 (0.0%)	0 (0.0%)	NA

Abbreviations: CI, confidence interval; DVT, deep‐vein thrombosis; ITT, intent‐to‐treat; NA, not applicable; PE, pulmonary embolism; VTE, venous thromboembolism.

^a^ Data shown are for all 273 patients with pancreatic cancer randomly assigned in the ITT population. All events were adjudicated by a blinded independent committee.

^b^ *P* = .328 (log‐rank test).

^c^ *P* = .034 (log‐rank test).

^d^ Numbers in the symptomatic and asymptomatic rows correspond to the number of patients who had any one of the symptomatic or asymptomatic events, respectively.

Additional benefit was observed with rivaroxaban when including secondary thrombotic efficacy endpoints. A composite of the primary endpoint with the addition of arterial and visceral thromboembolic events during the intervention period showed 6/135 (4.4%) events occurred in the rivaroxaban group, compared with 17/138 (12.3%) in the placebo (HR = 0.34; 95% CI, 0.14‐0.87; *P* = .02; NNT = 13). The composite efficacy endpoint that included symptomatic VTE and VTE‐related deaths occurred in 1/135 (0.7%) patients on rivaroxaban and 6/138 (4.3%) patients on placebo (HR = 0.16; 95% CI, 0.02‐1.37; *P* = .057) during the intervention period. For the secondary efficacy endpoint of all‐cause mortality, there were 11 (8.2%) deaths in the rivaroxaban group compared with 5 (3.6%) in the placebo group (HR = 2.09; 95% CI, 0.73‐6.02; *P* = .16) during the intervention period. During the up‐to‐day‐180 period, there were 34 (25.2%) deaths in the rivaroxaban group and 33 (23.9%) in the placebo group (HR = 1.05; 95% CI, 0.65‐1.69; *P* = .85). The cause of death was due to disease progression in most of the patients; 31/34 (91.2%) in the rivaroxaban group and 30/33 (90.9%) in the placebo group. There were no VTE‐related deaths in either treatment group during the intervention period. There was one patient (0.7%) with a fatal PE in the placebo group (occurred 25 days after end of treatment).

### 3.3 Safety outcomes

The primary safety endpoint of major bleeding occurred in 2/130 (1.5%) patients on rivaroxaban compared with 3/131 (2.3%) on placebo (HR = 0.67; 95% CI, 0.11‐3.99; *P* = .654; Table S1). Clinically relevant non‐major bleeding occurred in 5 (3.9%) patients on rivaroxaban and 2 (1.5%) patients on placebo (HR = 2.47; 95% CI, 0.48‐12.72; *P* = .264). There was no fatal bleeding event. The incidence of adverse events and serious adverse events was comparable between the two groups (Table S2).

### 3.4 Correlative biomarker studies

**Figure 2:**
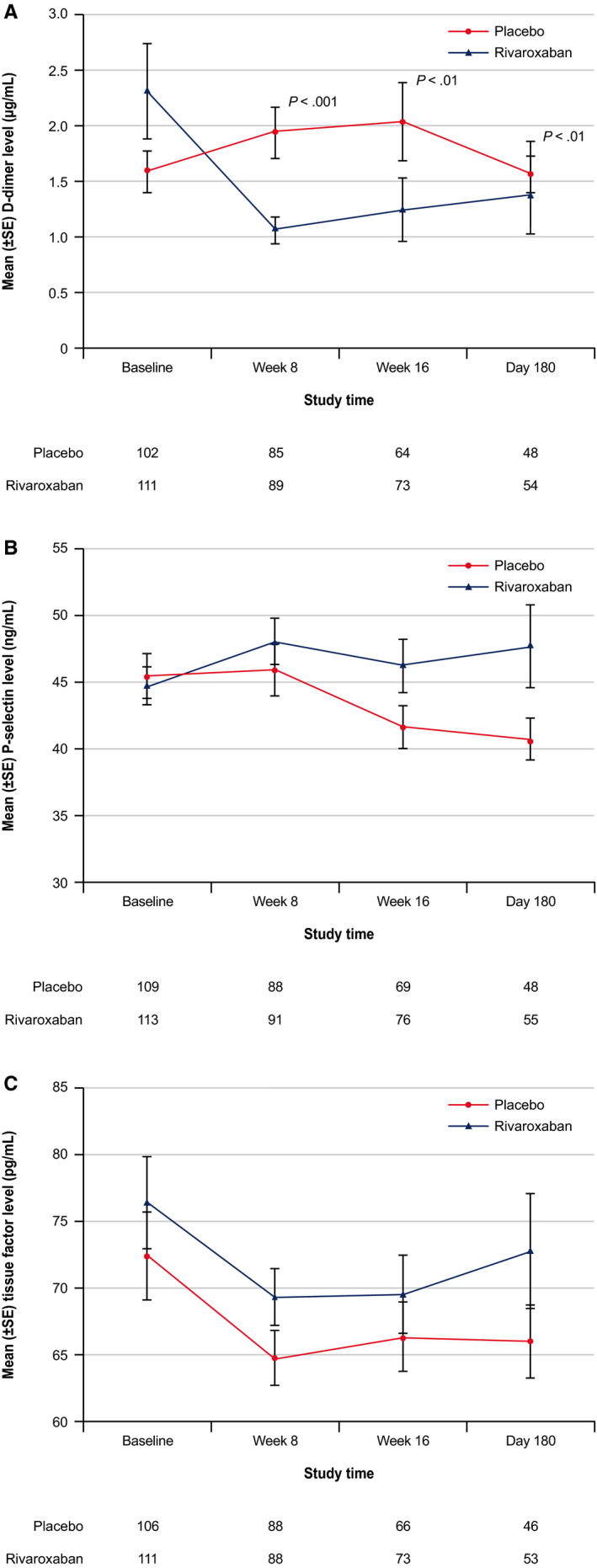
Effects of rivaroxaban vs placebo treatment on (A) D‐dimer levels, (B) tissue factor levels, and (C) P‐selectin levels during study among participants who did not experience a venous thromboembolism.^a^ SE, standard error. ^a^ *P*‐value based on Wilcoxon rank sum test. Safety population (randomly assigned and treated) was used and only the laboratory values during the treatment period were included for these summaries

To evaluate the biological effects of treatment on biomarkers associated with thrombosis, the levels of D‐dimer, P‐selectin, and TF were summarized. At baseline, the levels of these biomarkers were not significantly different between the treatment arms (Table 1). During the intervention period, in participants who had not experienced VTE, the rivaroxaban group showed significantly lower D‐dimer values over time (week 8, *P* < .001; week 16 and day 180, *P* < .01) compared with those on placebo (Figure 2); the D‐dimer values for all patients were also significantly lower for rivaroxaban (weeks 8 and 16; *P* < .001; day 180, *P* < .01) compared with placebo. The changes in P‐selectin and TF were not significantly different between treatment arms.

### 3.5 Analysis of predictors of VTE and survival

To determine which patients are at greater risk for VTE and therefore likely to benefit from thromboprophylaxis, univariate analysis of the clinical and biomarker data was performed (Tables S3 and S4). The potential risk factors included were clinical (age, gender, ECOG PS), stage of the disease, chemotherapy regimen (5‐fluorouracil–based or gemcitabine‐based regimen), components of the Khorana risk score (hemoglobin, leukocyte, platelet counts, and body mass index criteria), and baseline D‐dimer biomarker data. None of the variables tested were significantly associated with VTE on univariate analysis, other than thromboprophylaxis with rivaroxaban (*P* = .043) associated with lower risk for VTE during the intervention period (Tables S3 and S4).

To evaluate the effect of treatment on all‐cause mortality, the same risk factors were considered and univariate Cox proportional hazards models were fitted for each risk factor (Table 3 and Table S5). A number of risk factors, including ECOG PS ≥2, advanced‐stage disease (stage IV), Khorana risk score >2 and individual components of the Khorana risk score (anemia, elevated leukocytes, and platelets), elevated baseline D‐dimer levels, and chemotherapy type, were significantly associated with all‐cause mortality for the day 180 observation period, based on the univariate analysis (Table 3).

**Table 3:** Cox proportional hazards model for the risk of all‐cause mortality (ITT analysis population)

Univariate analysis	Up‐to‐day‐180 observation period		Intervention period	
Variables	Hazard ratio (95% CI)	*P*‐value	Hazard ratio (95% CI)	*P*‐value
Treatment (rivaroxaban 10 mg vs placebo)	1.05 (0.65‐1.69)	.848	2.09 (0.73‐6.02)	.172
Age	1.00 (0.98‐1.03)	.726	1.00 (0.95‐1.05)	.936
Gender (male vs female)	1.48 (0.90‐2.44)	.124	1.05 (0.39‐2.82)	.922
ECOG PS (≥2 vs <2)	5.29 (2.61‐10.71)	< .0001	11.59 (3.68‐36.50)	< .0001
Stage of cancer (stage III vs II)	0.82 (0.27‐2.55)	.735	0.37 (0.03‐4.07)	.415
Stage of cancer (stage IV vs II)	2.41 (1.03‐5.59)	.041	1.59 (0.36‐7.06)	.540
Baseline D‐dimer^a^ (>0.9 μg/mL vs ≤0.9 μg/mL)	2.22 (1.32‐3.72)	.002	2.45 (0.87‐6.90)	.089
Chemotherapy (5‐FU–based vs gemcitabine‐based)	0.58 (0.36‐0.96)	.033	0.31 (0.10‐0.97)	.045
Baseline Khorana risk score (>2 vs =2)	2.47 (1.53‐4.00)	< .0001	4.82 (1.75‐13.27)	.002
Individual components of the Khorana risk score (criteria present/not present at baseline)				
Hemoglobin (<10 g/dL vs not)	3.23 (1.59‐6.52)	.001	3.69 (0.83‐16.44)	.087
Leukocyte counts (>11 000/mm^3^ vs not)	2.38 (1.27‐4.44)	.007	5.15 (1.79‐14.84)	.002
Platelet counts (>350 000/mm^3^ vs not)	1.83 (1.04‐3.21)	.035	3.54 (1.28‐9.73)	.014
BMI (>35 kg/m^2^ vs not)	1.24 (0.45‐3.39)	.682	NA	NA^c^
Multivariable analysis^b^				
Variables				
Treatment (rivaroxaban 10 mg vs placebo)	1.15 (0.71‐1.86)	.578	2.29 (0.79‐6.67)	.129
ECOG PS (≥2 vs <2)	6.71 (3.22‐13.95)	< .0001	19.17 (5.48‐67.03)	< .0001
Hemoglobin (<10 g/dL vs not)	3.13 (1.51‐6.48)	.002		
Platelet counts (>350 000/mm^3^ vs not)	1.88 (1.04‐3.38)	.036	4.88 (1.66‐14.35)	.004

Abbreviations: 5‐FU, 5‐fluorouracil; BMI, body mass index; CI, confidence interval; ECOG PS, Eastern Cooperative Oncology Group performance status; ITT, intent‐to‐treat; NA, not applicable.

^a^ Baseline D‐dimer 75th percentile value = 0.9 μg/mL.

^b^ Multivariable models: Only significant predictors (*P* < .05) at univariate analysis were included in the model. Treatment was retained in the model even though it was not significant in the univariate analysis as it is a key factor in this study that describes the overall outcome that not only impacts the outcome directly but also mediates the association with outcome through the presence of other baseline characteristics. *P*‐values are not adjusted for multiple comparisons. Only the individual components of the total Khorana risk score were included in the model to evaluate which component of the risk score was a contributing factor.

^c^ There were no subjects with all‐cause mortality among the 14 subjects who had a BMI component present.

The results of the multivariable analysis for the up‐to‐day‐180 and intervention observation periods are shown in Table 3. In the final model for the day 180 observation period, only the ECOG PS (≥2), hemoglobin (<10 g/dL), and platelet count (≥350,000/mm^3^) were significant risk factors of mortality. Only the ECOG PS and platelet count remained significant in the multivariable model for the intervention period analysis.

## 4 DISCUSSION

The results of this study demonstrated that with rivaroxaban thromboprophylaxis in ambulatory pancreatic cancer patients initiating a new systemic cancer treatment, the difference in VTE rate or VTE‐related death, although lower, was not significant during the 180‐day study period. However, rivaroxaban significantly reduced the risk for VTE during the intervention period, without increasing the risk of major bleeding, as compared with placebo. An absolute difference of 6.4% was found in the primary composite endpoint of VTE and VTE‐related death (10.1% vs 3.7%), in favor of rivaroxaban over placebo during the intervention period. This absolute difference in VTE risk was observed despite excluding 6.6% of the enrolled patients who were found to have DVT at baseline screening, which is not routine practice, and may underestimate the benefit of thromboprophylaxis.

In our study, the symptomatic VTE events or VTE‐related deaths were lower during the intervention period with rivaroxaban (0.7% vs 4.3%; *P* = .057), as compared with placebo, which resulted in a relative risk reduction of 84%. These findings are consistent with prior studies^7^, ^8^, ^12^, ^13^; the meta‐analysis of four of these randomized clinical trials of LMWH in patients with advanced pancreatic cancer^14^ showed 82% reduction in the relative risk of symptomatic VTE, without an increase in major bleeding. The magnitude of VTE risk reduction seen in our trial is also similar to the results seen with the ultra‐LMWH semuloparin in the pancreatic cancer subset of the SAVE‐ONCO trial.^8^ The risk of major bleeding was also low in our trial, thus providing the necessary balance of benefit and harm in this setting. However, we recognize that the required BLE CUS at baseline and regular intervals during the study does not reflect clinical practice and may have introduced unintended bias into the data.

To determine which patients are at the greatest risk for VTE and would benefit from thromboprophylactic treatment, we examined known clinical and biomarker‐based risk factors. The analysis of the baseline clinical and biomarker‐based risk factors in this study did not identify any risk factors to be predictive of VTE in this population, other than thromboprophylaxis with rivaroxaban associated with a lower risk during the intervention period. Elevated levels of biomarkers, such as D‐dimers, have been reported as a risk factor predictive of VTE in patients with pancreatic cancer starting initial chemotherapy^14^; however, D‐dimer did not reach significance (*P* = .06) in predicting VTE in this study. This may be due to the study population, which included patients who received prior chemotherapy regimens and had a history of VTE.

The all‐cause mortality was not significantly different with rivaroxaban compared with placebo. In most of the cases, the cause of death was attributed to progression of the disease; poor performance status (ECOG PS ≥2), and more advanced disease (receiving a second line of treatment) may also have contributed to this. Furthermore, univariate and multivariable analysis of risk factors predictive of all‐cause mortality during the 180‐day study period showed pre‐treatment ECOG PS ≥2, platelet counts (≥350,000/mm^3^), and anemia (hemoglobin <10 g/dL) to be risk factors.

In patients who had not experienced VTE, the correlative D‐dimer values in the rivaroxaban group showed a significant decline during the study period compared to baseline, and compared to the placebo group in which the D‐dimer levels increased during the study period. D‐dimer levels have been shown to be an important prognostic factor; high levels have been found in patients with VTE and have been found to predict the recurrence of VTE.^15^, ^16^ Furthermore, high D‐dimer levels have been associated with poor survival in patients with various malignancies, including pancreatic cancer.^17^, ^18^

In summary, our findings should inform guideline recommendations for high‐risk patients, given the favorable risk‐benefit ratio and convenience of the oral route of administration.

## CONFLICT OF INTEREST

Saroj Vadhan‐Raj reports receiving personal fees for serving on a steering committee and non‐financial support for travel from Janssen, and her institution received grant support from Janssen for the conduct of the study. Outside the submitted work, Dr Vadhan‐Raj also reports receiving personal fees and non‐financial support for travel from Amgen, Sandoz, Shinogi, Tesaro, Enzychem, Dara, and Hospira, and grants to her institution from Amgen, Tesaro, Eli Lilly, American Regent, and Sanofi‐Aventis. Mairéad G. McNamara reports receiving research funding from NuCana, Shire, and Ipsen; speaker honoraria from NuCana and Mylan; and travel and accommodation support from Ipsen. Marino Venerito reports receiving honoraria from Nordic Pharma, Merck Serono, Bayer Vital, Lilly, and Sirtex and is a member of the advisory boards of Ipsen, Lilly, Nordic Pharma, BMS, MSD, Eisai, and Amgen. Hanno Riess reports receiving reimbursement for travel from Janssen Scientific Affairs during the conduct of the study and personal fees for serving on an advisory board from Janssen Scientific Affairs. Outside the submitted work, Dr Riess reports receiving personal fees for lectures and serving on advisory boards for Bayer, Boehringer Ingelheim, and Daiichi Sankyo; for lectures for BMS; for serving on an advisory board for Pfizer; and reports receiving grant support from Charité IIT. Eileen M. O’Reilly reports receiving grant support and personal fees from Janssen during the conduct of the study. Outside the submitted work, she reports receiving grant support from Acta Biologica, Agios, Array, AstraZeneca, Bayer, BeiGene, BMS, CASI, Celgene, Exelixis, Genentech, Halozyme, Incyte, Lilly, MabVax, Novartis, OncoQuest, Polaris Puma, QED, and Roche and personal fees from 3DMed, Agios, AlignMed, Amgen, Antengene, Aptus, ASLAN, Astellas, AstraZeneca, Bayer, BeiGene, Bioline, BMS, Boston Scientific, Bridgebio, CARsgen, Celgene, CASI, Cipla, CytomX, Daiichi, Debio, Delcath, Eisai, Exelixis, Genoscience, Gilead, Halozyme, Hengrui, Incyte, Inovio, Ipsen, Jazz, Janssen, Kyowa Kirin, LAM, Lilly, Loxo, Merck, Mina, NewLink Genetics, Novella, Onxeo, PCI Biotech, Pfizer, PharmaCyte, Pharmacyclics, Pieris, QED, RedHill, Sanofi, Servier, Silenseed, Sillajen, Sobi, Targovax, Tekmira, twoXAR, Vicus, Yakult, and Yiviva. Michael J. Overman reports receiving research funding from Bayer. Xiao Zhou reports receiving research funding from Janssen, Amgen, and TerSera. Ujjwala Vijapurkar reports being employed by Janssen Pharmaceuticals, Inc, and owning stock in Johnson & Johnson. Simrati Kaul reports being employed by Janssen Pharmaceuticals, Inc, and owning stock in Johnson & Johnson. She is also involved in the XARELTO development program at Janssen. Peter Wildgoose reports being employed by Janssen Scientific Affairs and owning stock in Johnson & Johnson. Alok A. Khorana reports receiving personal fees for serving as co‐chair of the steering committee for CASSINI and non‐financial support for travel from Janssen during the conduct of the study; personal fees and non‐financial support for travel from Bayer, Sanofi, Parexel, Janssen, Halozyme, Pfizer, AngioDynamics, Leo Pharma, Medscape/WebMD, and Seattle Genetics; personal fees from Pharmacyclics, Pharmacyte, and TriSalus; and grants to his institution from Merck, Array, BMS, and Leap Pharma, outside the submitted work. Dr Khorana was also the National Coordinator of the MARINER trial for Janssen.

## AUTHOR CONTRIBUTIONS

Saroj Vadhan‐Raj: Conceptualization, data curation, formal analysis, investigation, methodology, project administration, resources, supervision, writing—original draft, review and editing. Mairéad G. McNamara, Marino Venerito, Hanno Riess, Eileen M. O’Reilly, Michael J. Overman, Alok A. Khorana: Conceptualization, data curation, investigation, methodology, and writing—review and editing. Xiao Zhou and Ujjwala Vijapurkar: Data curation, formal analysis, and writing—review and editing. Simrati Kaul: Conceptualization, methodology, project administration, resources, software, supervision, and writing—review and editing.

## Supporting information

Supplementary Material

## Data Availability

At present, the sponsor's policy is to share data after regulatory approval for the specific indication evaluated in the study, in accordance with the policy of its codevelopment partner. Interested researchers can use www.clinicalstudydatarequest.com to request access to anonymized patient‐level data and supporting documents from clinical studies to conduct further research that can help advance medical science or improve patient care. Information on the criteria for listing studies and other relevant information is provided in the codevelopment partner's section of the portal.
