# Supplementary material for: Rivaroxaban thromboprophylaxis in ambulatory patients with pancreatic cancer: Results from a pre‐specified subgroup analysis of the randomized CASSINI study
Source: Cancer Med. 2020 Jul 14;9(17):6196–204. doi: 10.1002/cam4.3269 (PMC7476843; doi:10.1002/cam4.3269)
Supplement: Supplementary file 1 — Supplementary Material [file CAM4-9-6196-s001.docx]

**Supporting Information (Supplementary Appendix)**

**Figure S1. CONSORT diagram.**


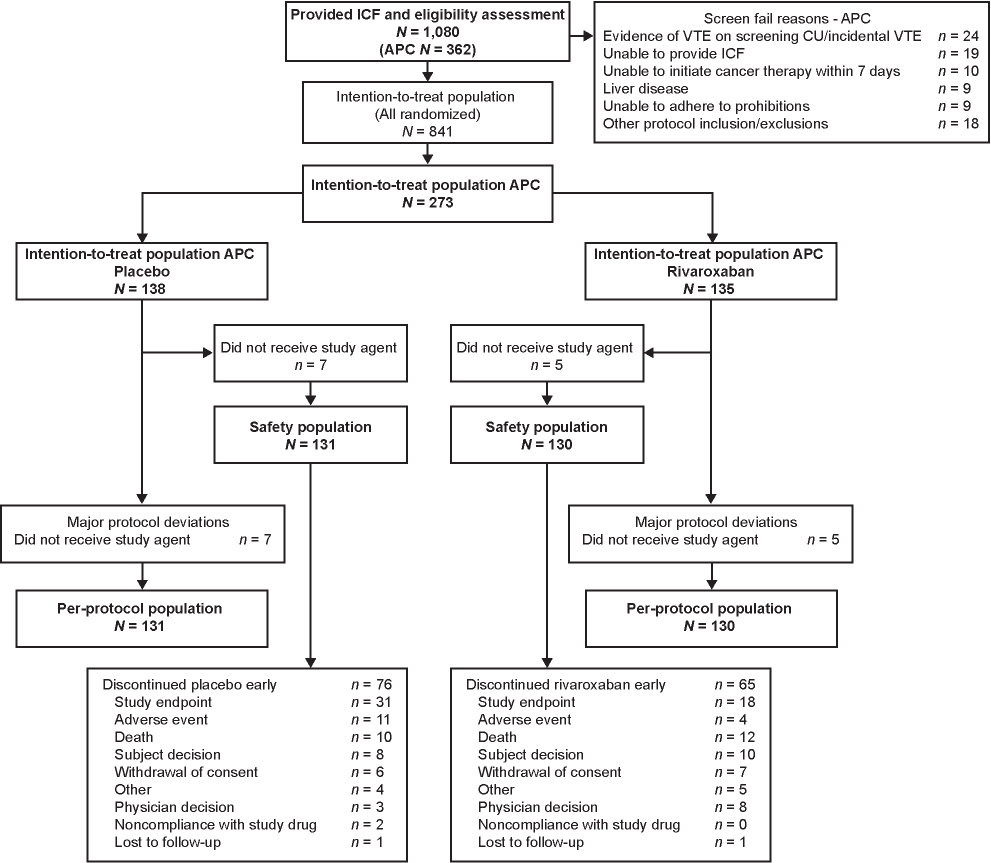


CONSORT, Consolidated Standards of Reporting Trials; ICF, informed consent form; APC, advanced pancreatic cancer**;** VTE, venous thromboembolism; CU, compression ultrasound.

**Table S1. Primary safety outcomes, according to treatment group^a^**

| **Patients, *n* (%)** | **Placebo**  **(*N*=131)** | **Rivaroxaban**  **(*N*=130)** | **Hazard ratio  (95% CI)** | ***P*-value** |
| --- | --- | --- | --- | --- |
| **Primary safety endpoint** |  |  |  |  |
| Major bleeding^b^ | 3 (2.3%) | 2 (1.5%) | 0.67 (0.11-3.99) | 0.654 |
| **Secondary safety endpoints** |  |  |  |  |
| Clinically relevant, non-major bleeding^c^ | 2 (1.5%) | 5 (3.9%) | 2.47 (0.48-12.72) | 0.264 |
| Any bleeding^d^ | 11 (8.4%) | 18 (13.8%) | 1.61 (0.76-3.41) | 0.21 |

CI, confidence interval.

^a^All bleeding events are defined based on the International Society on Thrombosis and Haemostasis classification.
^b^Major bleeding is defined as clinically overt bleeding that is associated with a reduction in hemoglobin of 2 g/dL or more, or a transfusion of two or more units of packed red blood cells or whole blood, or occurrence at a critical site defined as intracranial, intraspinal, intraocular, pericardial, intra-articular, intramuscular with compartment syndrome, retroperitoneal, or fatal outcome.

^c^Clinically relevant non-major bleeding is defined as overt bleeding not meeting the criteria for major bleeding but associated with medical intervention, or unscheduled contact with a physician, or temporary cessation of study treatment, or discomfort such as pain, or impairment of activities of daily life.
^d^Any bleeding is defined as a composite of major bleeding, clinically relevant non-major bleeding, or minor bleeding.

**Table S2. Summary of adverse events^a^**

| **Patients, n (%)** | **Placebo**  **(*N*=131)** | **Rivaroxaban**  **(*N*=130)** |
| --- | --- | --- |
| Any adverse event | 104 (79.4%) | 96 (73.8%) |
| Serious adverse event | 65 (49.6%) | 66 (50.8%) |
| Any adverse event related to study drug^b^ | 6 (4.6%) | 11 (8.5%) |
| Any adverse event leading to permanent study drug discontinuation | 26 (19.8%) | 20 (15.4%) |
| Any adverse event with outcome of death | 35 (26.7%) | 35 (26.9%) |

^a^All adverse events reported for subjects in the safety analysis population at any time during the study. Outcome events, including deep-vein thrombosis, pulmonary embolism, myocardial infarction, stroke, arterial thromboembolism, venous thromboembolism, and bleeding (including intracranial hemorrhage) were excluded from adverse event/serious adverse event collection and reporting, regardless of seriousness or severity.
^b^Related to study agent, as determined by investigator: possibly related, probably related, and very likely related.

**Table S3. Primary thromboembolic endpoint events (ITT analysis population)**

|  | | **Up-to-day-180 observation period** | | **Intervention period** | |
| --- | --- | --- | --- | --- | --- |
|  | | **Placebo** | **Rivaroxaban** | **Placebo** | **Rivaroxaban** |
| VTE primary efficacy endpoint |  | 18/138 (13.0%) | 13/135 (9.6%) | 14/138 (10.1%) | 5/135 (3.7%) |
| Gender | Female | 8/61 (13.1%) | 8/56 (14.3%) | 6/61 (9.8%) | 2/56 (3.6%) |
|  | Male | 10/77 (13.0%) | 5/79 (6.3%) | 8/77 (10.4%) | 3/79 (3.8%) |
| Baseline ECOG PS | 0-1 | 17/131 (13.0%) | 12/126 (9.5%) | 13/131 (9.9%) | 4/126 (3.2%) |
|  | ≥2 | 1/6 (16.7%) | 1/9 (11.1%) | 1/6 (16.7%) | 1/9 (11.1%) |
| Stage of cancer | Stage I | 0/1 | 0/6 | 0/1 | 0/6 |
|  | Stage II | 3/19 (15.8%) | 1/22 (4.5%) | 3/19 (15.8%) | 1/22 (4.5%) |
|  | Stage III | 2/24 (8.3%) | 0/19 | 2/24 (8.3%) | 0/19 |
|  | Stage IV | 11/89 (12.4%) | 12/82 (14.6%) | 8/89 (9.0%) | 4/82 (4.9%) |
| Baseline D-dimer^a^ | > 0.9 μg/mL | 5/27 (18.5%) | 4/37 (10.8%) | 4/27 (14.8%) | 1/37 (2.7%) |
|  | ≤0.9 μg/mL | 13/100 (13.0%) | 8/90 (8.9%) | 10/100 (10.0%) | 4/90 (4.4%) |
| Chemotherapy | 5-FU–based | 12/67 (17.9%) | 8/63 (12.7%) | 8/67 (11.9%) | 2/63 (3.2%) |
|  | Gemcitabine-based | 4/61 (6.6%) | 4/61 (6.6%) | 4/61 (6.6%) | 3/61 (4.9%) |
|  | Gemcitabine + capecitabine/5-FU | 2/6 (33.3%) | 0/7 | 2/6 (33.3%) | 0/7 |
|  | Other cytotoxic | 0/2 | 0/3 | 0/2 | 0/3 |
|  | Other noncytotoxic | 0/1 | 1/1 (100.0%) | 0/1 | 0/1 |
| Baseline Khorana risk score | =2 | 14/100 (14.0%) | 10/96 (10.4%) | 12/100 (12.0%) | 3/96 (3.1%) |
|  | >2 | 4/38 (10.5%) | 3/39 (7.7%) | 2/38 (5.3%) | 2/39 (5.1%) |
| Individual components of the Khorana risk score (criteria present/not present at baseline) | | |  |  |  |
| Hemoglobin | Hemoglobin <10 g/dL | 1/7 (14.3%) | 1/8 (12.5%) | 1/7 (14.3%) | 0/8 |
|  | Not present | 17/131 (13.0%) | 12/127 (9.4%) | 13/131 (9.9%) | 5/127 (3.9%) |
| Platelets | Platelet count >350,000/mm^3^ | 3/20 (15.0%) | 1/22 (4.5%) | 2/20 (10.0%) | 0/22 |
|  | Not present | 15/118 (12.7%) | 12/113 (10.6%) | 12/118 (10.2%) | 5/113 (4.4%) |
| Leukocytes | Leukocyte count >11,000/mm^3^ | 1/15 (6.7%) | 2/11 (18.2%) | 0/15 | 1/11 (9.1%) |
|  | Not present | 17/123 (13.8%) | 11/124 (8.9%) | 14/123 (11.4%) | 4/124 (3.2%) |
| BMI | BMI >35 kg/m^2^ | 1/8 (12.5%) | 1/6 (16.7%) | 0/8 | 1/6 (16.7%) |
|  | Not present | 17/130 (13.1%) | 12/129 (9.3%) | 14/130 (10.8%) | 4/129 (3.1%) |

ITT, intent-to-treat; VTE, venous thromboembolism; ECOG PS, Eastern Cooperative Oncology Group performance status; 5-FU, 5-fluorouracil; BMI, body mass index.

^a^Baseline D-dimer 75th percentile value = 0.9 μg/mL.

**Table S4. Univariate Cox proportional hazards model for the risk of VTE (ITT analysis population)**

|  | **Up-to-day-180 observation period** | | **Intervention period** | |  |
| --- | --- | --- | --- | --- | --- |
| **Variables** | **Hazard ratio (95% CI)** | ***P*-value** | **Hazard ratio (95% CI)** | ***P*-value** | |
| Treatment (rivaroxaban 10 mg vs. placebo) | 0.70 (0.34-1.43) | 0.329 | 0.35 (0.13-0.96) | 0.043 | |
| Age | 1.00 (0.97-1.04) | 0.960 | 1.01 (0.96-1.06) | 0.673 | |
| Gender (M vs. F) | 0.77 (0.38-1.55) | 0.456 | 1.12 (0.45-2.77) | 0.815 | |
| ECOG PS (≥2 vs. ˂2) | 2.43 (0.58-10.24) | 0.227 | 3.96 (0.91-17.21) | 0.066 | |
| Stage of cancer (Stage III vs. II) | 0.42 (0.08-2.30) | 0.319 | 0.38 (0.07-2.07) | 0.263 | |
| Stage of cancer (Stage IV vs. II) | 1.55 (0.54-4.49) | 0.417 | 0.75 (0.24-2.31) | 0.611 | |
| Baseline D-dimer^a^ (>0.9 μg/mL vs. ≤0.9 μg/mL) | 1.52 (0.70-3.33) | 0.291 | 1.32 (0.47-3.66) | 0.596 | |
| Chemotherapy (5-FU–based vs. gemcitabine-based) | 2.11 (0.93-4.79) | 0.075 | 1.20 (0.46-3.16) | 0.708 | |
| Baseline Khorana risk score (>2 vs. =2) | 0.84 (0.36-1.96) | 0.693 | 0.78 (0.26-2.36) | 0.665 | |
| Individual components of the Khorana risk score (criteria present/not present at baseline) |  |  |  |  | |
| Hemoglobin (<10 g/dL vs. not) | 1.46 (0.35-6.12) | 0.607 | 1.39 (0.18-10.45) | 0.752 | |
| Leukocyte counts (>11,000/mm^3^ vs. not) | 1.10 (0.33-3.60) | 0.881 | 0.62 (0.08-4.61) | 0.636 | |
| Platelet counts (>350,000/mm^3^ vs. not) | 0.83 (0.29-2.38) | 0.733 | 0.69 (0.16-3.00) | 0.625 | |
| BMI (>35 kg/m^2^ vs. not) | 1.64 (0.39-6.87) | 0.501 | 1.42 (0.19-10.64) | 0.733 | |

VTE, venous thromboembolism; ITT, intent-to-treat; CI, confidence interval; M, male; F, female; ECOG PS, Eastern Cooperative Oncology Group performance status; 5-FU, 5-fluorouracil; BMI, body mass index.

*P*-values are not adjusted for multiple comparisons.

^a^Baseline D-dimer 75^th^ percentile value = 0.9 μg/mL.

**Table S5. All-cause mortality endpoint events (ITT analysis population)**

|  | | **Up-to-day-180 observation period** | | **Intervention period** | |
| --- | --- | --- | --- | --- | --- |
|  | | **Placebo** | **Rivaroxaban** | **Placebo** | **Rivaroxaban** |
| All-cause mortality efficacy endpoint |  | 33/138 (23.9%) | 34/135 (25.2%) | 5/138 (3.6%) | 11/135 (8.1%) |
| Gender | Female | 9/61 (14.8%) | 15/56 (26.8%) | 1/61 (1.6%) | 6/56 (10.7%) |
|  | Male | 24/77 (31.2%) | 19/79 (24.1%) | 4/77 (5.2%) | 5/79 (6.3%) |
| Baseline ECOG PS | 0-1 | 31/131 (23.7%) | 27/126 (21.4%) | 5/131 (3.8%) | 7/126 (5.6%) |
|  | ≥2 | 2/6 (33.3%) | 7/9 (77.8%) | 0/6 | 4/9 (44.4%) |
| Stage of cancer | Stage I | 0/1 | 0/6 | 0/1 | 0/6 |
|  | Stage II | 4/19 (21.1%) | 2/22 (9.1%) | 1/19 (5.3%) | 1/22 (4.5%) |
|  | Stage III | 5/24 (20.8%) | 1/19 (5.3%) | 0/24 | 1/19 (5.3%) |
|  | Stage IV | 23/89 (25.8%) | 31/82 (37.8%) | 4/89 (4.5%) | 9/82 (11.0%) |
| Baseline D-dimer | >0.9 μg/mL | 10/27 (37.0%) | 14/37 (37.8%) | 2/27 (7.4%) | 4/37 (10.8%) |
|  | ≤0.9 μg/mL | 19/100 (19.0%) | 17/90 (18.9%) | 2/100 (2.0%) | 7/90 (7.8%) |
| Chemotherapy | 5-FU–based | 14/67 (20.9%) | 13/63 (20.6%) | 2/67 (3.0%) | 2/63 (3.2%) |
|  | Gemcitabine-based | 19/61 (31.1%) | 19/61 (31.1%) | 3/61 (4.9%) | 8/61 (13.1%) |
|  | Gemcitabine + capecitabine/5-FU | 0/6 | 1/7 (14.3%) | 0/6 | 1/7 (14.3%) |
|  | Other cytotoxic | 0/2 | 0/3 | 0/2 | 0/3 |
|  | Other noncytotoxic | 0/1 | 1/1 (100.0%) | 0/1 | 0/1 |
| Baseline Khorana risk score | =2 | 18/100 (18.0%) | 18/96 (18.8%) | 2/100 (2.0%) | 4/96 (4.2%) |
|  | >2 | 15/38 (39.5%) | 16/39 (41.0%) | 3/38 (7.9%) | 7/39 (17.9%) |
| Individual components of the Khorana risk score (criteria present/not present at baseline) | | |  |  |  |
| Hemoglobin | Hemoglobin <10 g/dL | 5/7 (71.4%) | 4/8 (50.0%) | 1/7 (14.3%) | 1/8 (12.5%) |
|  | Not present | 28/131 (21.4%) | 30/127 (23.6%) | 4/131 (3.1%) | 10/127 (7.9%) |
| Platelets | Platelet count >350,000/mm^3^ | 8/20 (40.0%) | 8/22 (36.4%) | 2/20 (10.0%) | 4/22 (18.2%) |
|  | Not present | 25/118 (21.2%) | 26/113 (23.0%) | 3/118 (2.5%) | 7/113 (6.2%) |
| Leukocytes | Leukocyte count >11,000/mm^3^ | 5/15 (33.3%) | 7/11 (63.6%) | 2/15 (13.3%) | 3/11 (27.3%) |
|  | Not present | 28/123 (22.8%) | 27/124 (21.8%) | 3/123 (2.4%) | 8/124 (6.5%) |
| BMI | BMI >35 kg/m^2^ | 2/8 (25.0%) | 2/6 (33.3%) | 0/8 | 0/6 |
|  | Not present | 31/130 (23.8%) | 32/129 (24.8%) | 5/130 (3.8%) | 11/129 (8.5%) |

ITT, intent-to-treat; ECOG PS, Eastern Cooperative Oncology Group performance status; 5-FU, 5-fluorouracil; BMI, body mass index.
